# Supplementary material for: Single dose of intravenous miR199a-5p delivery targeting ischemic heart for long-term repair of myocardial infarction
Source: Nat Commun. 2024 Jul 2;15:5565. doi: 10.1038/s41467-024-49901-x (PMC11219733; doi:10.1038/s41467-024-49901-x)
Supplement: Supplementary file 1 — Supplementary Information [file 41467_2024_49901_MOESM1_ESM.pdf]

Supplementary Materials for

Single dose of intravenous miR199a-5p delivery targeting ischemic heart for long-term repair of myocardial infarction

Yu Chen *et al.*

*Corresponding author. Email: qqiuxzh@163.com, ss.hhh89@hotmail.com

This PDF file includes:

Figs. S1 to S5

Fig. S1.

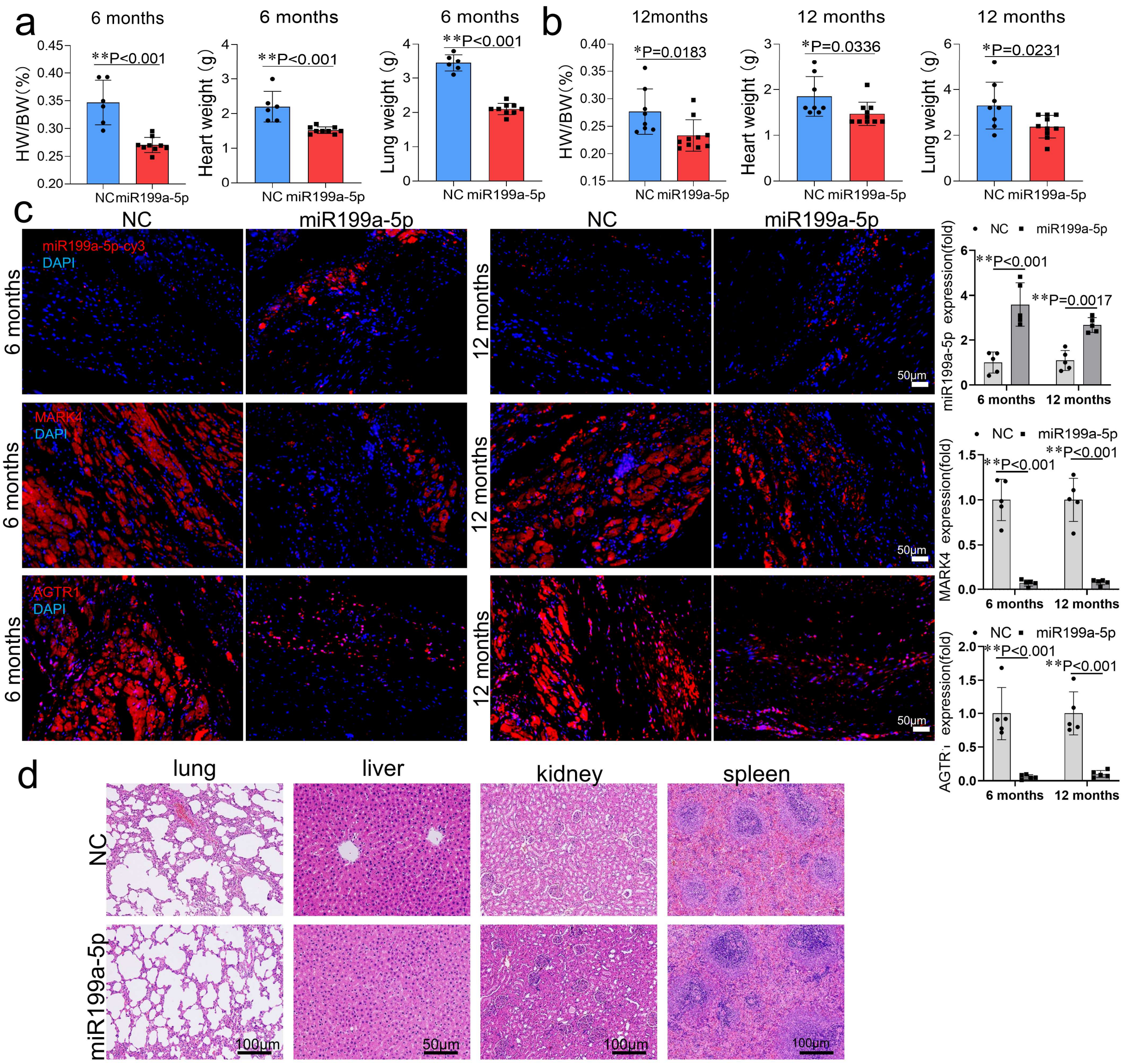

Fig.S1 In vivo safety assessment. Heart weigh/body weight, heart weight and lung weight of rats after miR199a-5p mimic injection at 6 months(**a**, n = 6 rats, mimic control; n = 9 rats, miR199a-5p mimics) and 12 months(**b**, n = 8 rats, mimic control; n = 10 rats, miR199a-5p mimics). **c.** The expression of miR199a-5p in the infarct zone was detected by FISH technique, and the expression of AGTR1 and MARK4 proteins in the infarct zone was detected by immunofluorescence, (n=5 rats). **d.** H&E staining results of major organs in rats injected with miR199a-5p or control mimic for 12 months. Data for figures S1a-c are presented as mean ± SD. The p-values were generated using an unpaired two-tailed Student's t-test. *P < 0.05, **P < 0.01.

Fig. S2.

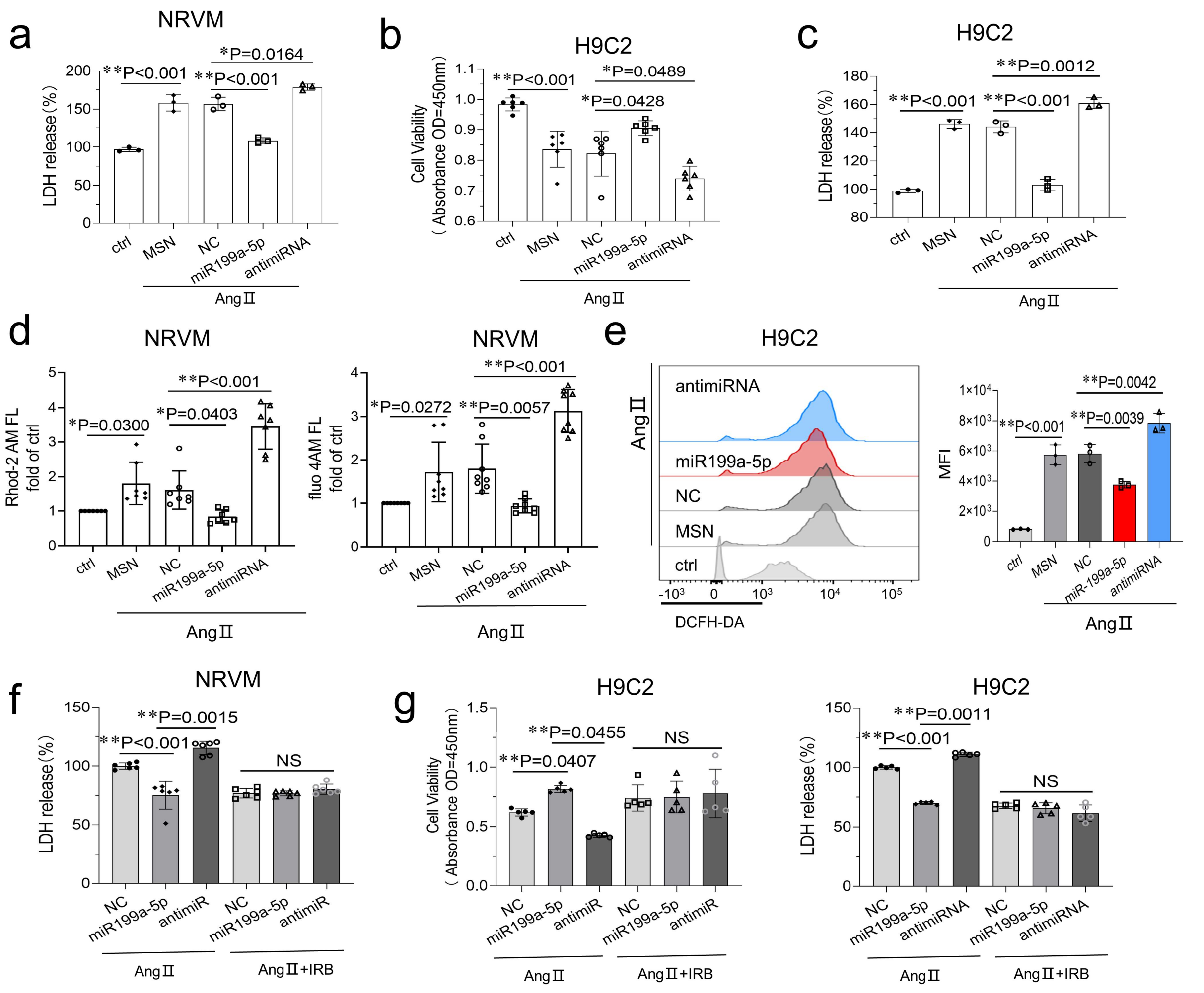

Fig.S2 miR199a-5p attenuates ROS and apoptosis via AGTR1. a-b. The cardiomyocytes were transduced with miR199a-5p or antimiR199a-5p for 1 h and then incubated with/without Ang II(0.1μM) for 48 hours. Supernatants were collected and cell viability was LDH assays, n=3 independent samples**(a)**. **b-c.** The H9C2 cells were transduced with miR199a-5p or antimiR199a-5p for 1 h and then incubated with/without Ang II(0.1μM) for 48 hours. Supernatants were collected and cell viability was detected by CCK-8**(b)**, n=6 and LDH assays, n=3 independent samples**(c)**. Relative cytosolic and mitochondrial calcium levels were assessed by Fura-2 fluorescence and Rhod-2 fluorescence in cardiomyocytes, n=7 independent samples(d). **e.** The H9C2 cells were transduced with miR199a-5p or antimiR199a-5p for 1 h and then incubated with/without Ang II(0.1μM) for 48 hours. Total intracellular ROS in DCFH-DA staining was detected by flow cytometry, n=3 independent samples. **f.** After transfecting with miR199a-5p or antimiR199a-5p for 1 h, NRVMs were cultured with Ang II and with or without irbesartan (IRB) for 48 hours. Detection of cell viability by LDH, n=6 independent samples. **g.** After transfecting with miR199a-5p or antimiR199a-5p for 1 h, H9C2 cells were cultured with Ang II and with or without IRB for 48 hours. Detection of cell viability by CCK-8 and LDH, n=5 independent samples. All data are presented as mean ± SD. of at least 3 independent experiments. The p-values were

generated by one-way analysis of variance (ANOVA), followed by Tukey's multiple-comparison post hoc test. *P < 0.05, **P < 0.01, NS, not significant.

Fig.S3.

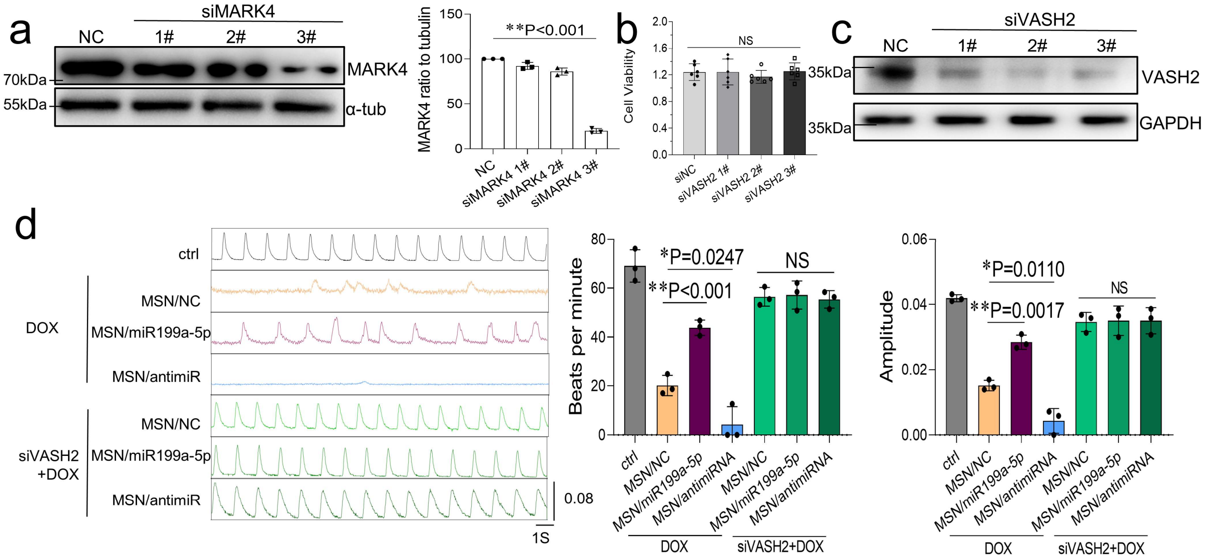

Fig.S3 miR199a-5p regulates cardiomyocyte contractility through VASH2. **a.** NRVMs were transfected with MARK4-targeting siRNAs (1#,2#,3#) for 48 hours. The expression level of MARK4 protein was assayed by immunoblotting, n=3 independent samples. **b-c.** NRVMs were transfected with VASH2-targeted siRNA (1#, 2#, 3#) for 48 h. Cell viability was detected by CCK-8, n=6 independent samples**(b),** The expression level of VASH2 protein was assayed by immunoblotting, n=3 independent samples**(c)**. **d.** After transfecting with VASH2-targeting siRNAs 2# or NC siRNA for 48 hours, NRVMs were transduced with miR199a-5p or antimiR199a-5p for 1 h and then incubated with DOX(1μM) within 24 hours. Label-free impedance analysis of spontaneously contracting NRVMs under different treatments using the RTCA cardio system. Representative images of NRVMs' real-time beating activity and quantification of its beating frequency and amplitude, n=3 independent samples. All data are presented as mean ± SD. of at least 3 independent experiments. The p-values were generated by one-way analysis of variance (ANOVA), followed by Tukey's multiple-comparison post hoc test. *P < 0.05, **P < 0.01, NS, not significant.

Fig. S4.

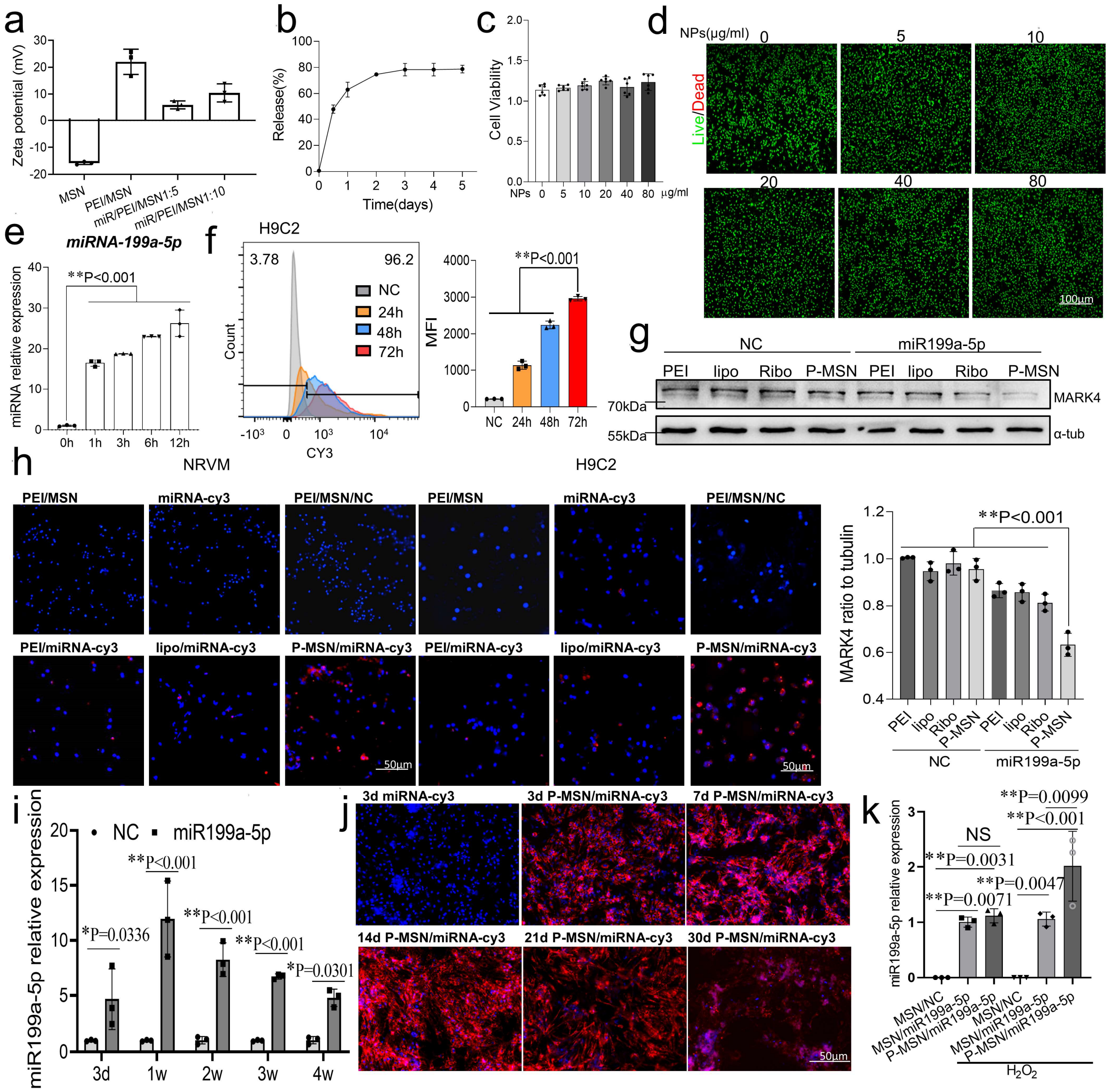

Fig.S4 The characterization of p-MSN/miRNA nanoparticles. a. Dynamic light scattering (DLS) measures the zeta potential of nanoparticles. **b.** Release profile of p-MSN/miRNA complexes releasing miRNA in vitro. Cell viability assay**(c)** and Live-dead staining**(d)** after transfection of cardiomyocytes with different concentrations of nanoparticles for 72 hours. **e.** After P-MSN/miR199a-5p transfection of NRVMs, RT-qPCR was performed to detect miR199a-5p expression within the indicated time points. **f.** Representative flow cytometry analysis of cy3-miR199a-5p transfection in H9C2 cells after 72 h of incubation with P-MSN/miR199a-5p nanocarriers, n=3 independent samples. **g.** WB detection of protein expression of MARK4 in transfected cardiomyocytes 72 hours after miR199a-5p packaging by different transfection reagents, n=3 independent sample. **h.** Fluorescence microscopy images of different transfection reagents coated with miRNA-cy3 after 8 hours of transfection in cardiomyocytes (left panel) and

H9C2 cells (right panel). **i.** RT-qPCR was assayed for miR199a-5p expression in cardiomyocytes within 30 days of P-MSN /miR199a-5p transfection, n=3 independent sample. **j.** Fluorescence microscopy image of P-MSN-encapsulated miRNA-cy3 transfected cardiomyocytes within 30 days. **k.** RT-qPCR analysis of miR199a-5p expression in MSN/miR199a-5p or P-MSN/miR199a-5p-treated normal or oxidatively injured cardiomyocytes treated with H_2O_2, n=3 independent sample. Quantitative data were expressed as the mean ± SD. of at least 3 independent experiments. The p-values for figure S4i was generated using an unpaired two-tailed Student's t-test, p value for figures S4c, e, g, k were generated by one-way analysis of variance (ANOVA), followed by Tukey's multiple-comparison post hoc test. $^*P < 0.05$, $^{**}P < 0.01$, NS, not significant.

Fig. S5.

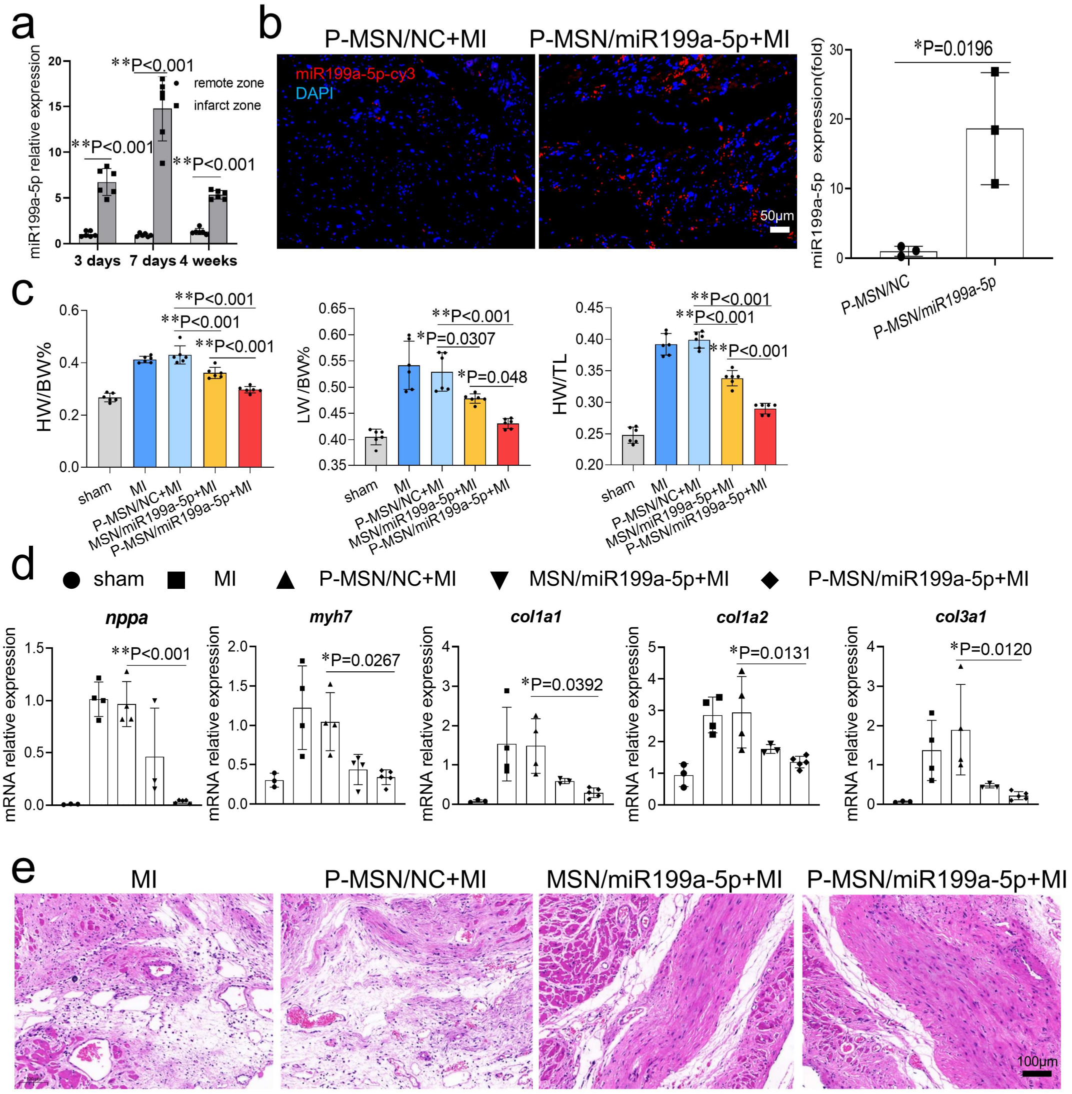

Fig.S5 P-MSN /miR199a-5p nanoparticles repair post-infarction cardiac function. a. 4 weeks after tail vein injection of P-MSN/miR199a-5p nanoparticles in MI rats, RT-qPCR was performed to detect miR199a-5p expression in cardiac infarcted and non-infarcted regions, n = 6 rats. **b.** FISH technique to detect miR199a-5p expression in the infarcted area of rat hearts 4 weeks after tail vein

injection of P-MSN/ miR199a-5p nanoparticles, n=3 rats. **c.** Heart weight/body weight (HW/BW) ratio, lung weight/body weight (LW/BW) ratio and heart weight/tibial length (HW/TL) ratio of rats after P-MSN/miR199a-5p nanoparticles injection at 4 weeks, n=6 rats. **d.** RT-qPCR detection of expression of pathological remodeling marker genes and fibrotic remodeling marker genes 4 weeks after P-MSN/miR199a-5p nanoparticle injection and MI injury. n = 5 rats. **e.** H&E staining results of infarcted area in rats herat injected with infarcted area for 4 weeks, n=3 rats. All data are presented as mean ± SD. The p-values for figures S4a, b were generated using an unpaired two-tailed Student's t-test, p value for figures S4c, d were generated by one-way analysis of variance (ANOVA), followed by Tukey's multiple-comparison post hoc test. $*P < 0.05$, $**P < 0.01$, NS, not significant.
