# Supplementary material for: Single dose of intravenous miR199a-5p delivery targeting ischemic heart for long-term repair of myocardial infarction
Source: Nat Commun. 2024 Jul 2;15:5565. doi: 10.1038/s41467-024-49901-x (PMC11219733; doi:10.1038/s41467-024-49901-x)
Supplement: Supplementary file 3 — Description of Additional Supplementary Files [file 41467_2024_49901_MOESM3_ESM.doc]

Description of Additional Supplementary Files

File Name: Supplementary Data 1.

Description:The echocardiographic data of the rat in Figure 2c.

File Name: Supplementary Data 2.

Description:The echocardiographic data of the rat in figure 7f.

File Name: Supplementary Data 3.

Description:Sequences of primers.
