# Supplementary material for: Single dose of intravenous miR199a-5p delivery targeting ischemic heart for long-term repair of myocardial infarction
Source: Nat Commun. 2024 Jul 2;15:5565. doi: 10.1038/s41467-024-49901-x (PMC11219733; doi:10.1038/s41467-024-49901-x)
Supplement: Supplementary file 7 — Reporting Summary [file 41467_2024_49901_MOESM7_ESM.pdf]

nature portfolio

Corresponding author(s): Xiaozhong Qiu, Honghao Hou

Last updated by author(s): Jun 17, 2024

Reporting Summary

Nature Portfolio wishes to improve the reproducibility of the work that we publish. This form provides structure for consistency and transparency in reporting. For further information on Nature Portfolio policies, see our Editorial Policies and the Editorial Policy Checklist.

Statistics

For all statistical analyses, confirm that the following items are present in the figure legend, table legend, main text, or Methods section.

n/a	Confirmed	
☐	☒	The exact sample size (*n*) for each experimental group/condition, given as a discrete number and unit of measurement
☐	☒	A statement on whether measurements were taken from distinct samples or whether the same sample was measured repeatedly
☐	☒	The statistical test(s) used AND whether they are one- or two-sided<br>*Only common tests should be described solely by name; describe more complex techniques in the Methods section.*
☒	☐	A description of all covariates tested
☐	☒	A description of any assumptions or corrections, such as tests of normality and adjustment for multiple comparisons
☐	☒	A full description of the statistical parameters including central tendency (e.g. means) or other basic estimates (e.g. regression coefficient) AND variation (e.g. standard deviation) or associated estimates of uncertainty (e.g. confidence intervals)
☐	☒	For null hypothesis testing, the test statistic (e.g. *F*, *t*, *r*) with confidence intervals, effect sizes, degrees of freedom and *P* value noted<br>*Give P values as exact values whenever suitable.*
☒	☐	For Bayesian analysis, information on the choice of priors and Markov chain Monte Carlo settings
☒	☐	For hierarchical and complex designs, identification of the appropriate level for tests and full reporting of outcomes
☒	☐	Estimates of effect sizes (e.g. Cohen's *d*, Pearson's *r*), indicating how they were calculated

Our web collection on statistics for biologists contains articles on many of the points above.

Software and code

Policy information about availability of computer code

Data collection	GEO database, miRBD, miRmap, tragetscan, and microT site, RTCA Cardio Software,Zeiss Zen software (ver. 3.2).
Data analysis	Graphpad Prism 8, SPSS version 23Image J v1.50, FlowJo 8.0

For manuscripts utilizing custom algorithms or software that are central to the research but not yet described in published literature, software must be made available to editors and reviewers. We strongly encourage code deposition in a community repository (e.g. GitHub). See the Nature Portfolio guidelines for submitting code & software for further information.

Data

Policy information about availability of data

All manuscripts must include a data availability statement. This statement should provide the following information, where applicable:

- Accession codes, unique identifiers, or web links for publicly available datasets
- A description of any restrictions on data availability
- For clinical datasets or third party data, please ensure that the statement adheres to our policy

Data that support the findings in this study are available from the corresponding author upon reasonable request.

Research involving human participants, their data, or biological material

Policy information about studies with human participants or human data. See also policy information about sex, gender (identity/presentation), and sexual orientation and race, ethnicity and racism.

Reporting on sex and gender	N/A
Reporting on race, ethnicity, or other socially relevant groupings	N/A
Population characteristics	N/A
Recruitment	N/A
Ethics oversight	N/A

Note that full information on the approval of the study protocol must also be provided in the manuscript.

Field-specific reporting

Please select the one below that is the best fit for your research. If you are not sure, read the appropriate sections before making your selection.

[x] Life sciences [ ] Behavioural & social sciences [ ] Ecological, evolutionary & environmental sciences

For a reference copy of the document with all sections, see nature.com/documents/nr-reporting-summary-flat.pdf

Life sciences study design

All studies must disclose on these points even when the disclosure is negative.

Sample size	Sample size were determined by similar studies, including animal (rat)studies, human studies and cell studies.
Data exclusions	No data was excluded for this study.
Replication	Unless specified, data were representative of three independent experiments. All the experiments and analyses shown could be successfully and reliably replicated and reproduced.
Randomization	All patient samples were randomly collected with control samples in the study. For animal study, all the animals were randomly divided into different groups for experiments.
Blinding	Pathological images were blind during scoring process.

Reporting for specific materials, systems and methods

We require information from authors about some types of materials, experimental systems and methods used in many studies. Here, indicate whether each material, system or method listed is relevant to your study. If you are not sure if a list item applies to your research, read the appropriate section before selecting a response.

Materials & experimental systems

n/a	Involved in the study	
[ ]	[x]	Antibodies
[ ]	[x]	Eukaryotic cell lines
[x]	[ ]	Palaeontology and archaeology
[ ]	[x]	Animals and other organisms
[x]	[ ]	Clinical data
[x]	[ ]	Dual use research of concern
[x]	[ ]	Plants

Methods

n/a	Involved in the study	
[x]	[ ]	ChIP-seq
[ ]	[x]	Flow cytometry
[x]	[ ]	MRI-based neuroimaging

Antibodies

Antibodies used	Angiotensin II Type 1 Receptor (AGTR1, Abcam,ab239995, WB recommended concentration: 1 μg/ml)<br>Cardiac troponin T (CTNT, Abcam,ab10214, IF recommended concentration: 1 μg/ml)<br>α-actinin (Abcam,ab9465, IF recommended concentration: 2 μg/ml)<br>8-OHG (Abcam,aab62623, IF recommended concentration: 2 μg/ml)

	Alpha Tubulin (α-tub, Abcam,ab7291, WB recommended concentration: 1 μg/ml)<br>NADPH oxidase 4 (NOX4, Abcam,ab154244, WB recommended concentration: 1 μg/ml)<br>Detyrosinated alpha Tubulin (dTyr-tub, Abcam,ab254154, WB recommended concentration: 1 μg/ml)<br>MARK4(Cell Signaling Technology,4834,WB recommended concentration: 1 μg/ml)
Validation	Anti-AGTR1 has been validated for use in western blotting, as stated on the abcam product page.<br>Anti-CTNT,has been validated for use in immunofluorescence, as stated on the abcam product page. This antibody was also validated in previous work (Li Y et al. Injectable hydrogel with MSNs/microRNA-21-5p delivery enables both immunomodification and enhanced angiogenesis for myocardial infarction therapy in pigs.sci Adv.2021).<br>Anti-α-actinin has been validated for use in western blotting, as stated on the abcam product page. This antibody was also validated in previous work (Li Z et al. Postnatal state transition of cardiomyocyte as a primary step in heart maturation. Protein Cell .2022).<br>Anti-8-OHG has been validated for use in immunofluorescence, as stated on the abcam product page. This antibody was also validated in previous work (Yan M et al. Mitochondrial damage and activation of the cytosolic DNA sensor cGAS-STING pathway lead to cardiac pyroptosis and hypertrophy in diabetic cardiomyopathy mice. Cell Death Discov.2022).<br>Anti-α-tub has been validated for use in western blotting, as stated on the abcam product page. This antibody was also validated in previous work (Seim GL et al. Nitric oxide-driven modifications of lipoic arm inhibit α-ketoacid dehydrogenases. Nat Chem Biol .2023).<br>Anti-NOX4 has been validated for use in western blotting, as stated on the abcam product page.<br>Anti-dTyr-tub, has been validated for use in western blotting, as stated on the abcam product page. This antibody was also validated in previous work (Estève MA et al. NBcl-2 down-regulation and tubulin subtype composition are involved in resistance of ovarian cancer cells to vinflunine. Mol Cancer Ther.2006).<br>Anti-MARK4 has been validated for use in western blotting, as stated on the Cell Signaling Technology product page. This antibody was also validated in previous work (Bo Yu, et al. KMT5A-methylated SNIP1 promotes triple-negative breast cancer metastasis by activating YAP signaling. Nat Commun.2022).

Eukaryotic cell lines

Policy information about cell lines and Sex and Gender in Research

Cell line source(s)	H9C2(CL-0089) and HEK 293T(CL-0005) cells were procured from Pricella Biotechnology (Wuhan, China).
Authentication	The main test for authenticating H9C2 cells and HEK 293T cells is short tandem repeat (STR) profiling, the analysis of microsatellite regions of DNA that have variable numbers of repeats and are located throughout the genome. PCR amplicons are generated using primers for regions that flank these polymorphic sites. After amplification, PCR products are resolved using capillary electrophoresis, and a profile for these repetitive regions is created.
Mycoplasma contamination	I confirmed that H9C2 cells and HEK293T cells tested negative for mycoplasma contamination.
Commonly misidentified lines<br>(See ICLAC register)	There were no misidentified cells in our studies.

Animals and other research organisms

Policy information about studies involving animals; ARRIVE guidelines recommended for reporting animal research, and Sex and Gender in Research

Laboratory animals	Eight-week old 220±20 g male S-D rats were purchased from the Animal Institute of Southern Medical University (Guangzhou, China).
Wild animals	This study did not involve wild animals.
Reporting on sex	Male S-D rats were used for animal experiments because experimental animals need to be observed for 1 year and menopause affects the cardiovascular system in female rats.
Field-collected samples	This study did not involve samples collected from the field.
Ethics oversight	All animal procedures were approved by the welfare and Ethical Committee for Experimental Animal Care of Southern Medical University.

Note that full information on the approval of the study protocol must also be provided in the manuscript.

Plants

Seed stocks	N/A
Novel plant genotypes	N/A
Authentication	N/A

Flow Cytometry

Plots

Confirm that:

- [x] The axis labels state the marker and fluorochrome used (e.g. CD4-FITC).
- [x] The axis scales are clearly visible. Include numbers along axes only for bottom left plot of group (a 'group' is an analysis of identical markers).
- [x] All plots are contour plots with outliers or pseudocolor plots.
- [x] A numerical value for number of cells or percentage (with statistics) is provided.

Methodology

Sample preparation	Primary cultured NRVMs were obtained from the hearts of S-D rats aged 1-3 days.After NRVMs and H9C2 cells were added to different treatments, 0.25% trypsin was added to digest the cells into single-cell suspensions, which were stained using Annexin V-FITC/PI or DCFH-DA probes and washed three times with PBS, and fluorescence intensities were measured with a FACScan instrument by BD Biosciences
Instrument	BD LSRFortessa X-20
Software	FlowJo 8.0
Cell population abundance	NRVMs:95%-97% purity,H9C2 cells were used in experiments,and the cell purity was identified by STR Authentication.
Gating strategy	Unstained cells are used as the negative control of the experiment.

- [x] Tick this box to confirm that a figure exemplifying the gating strategy is provided in the Supplementary Information.
